# Gut microbiome phytoestrogen production and its protective role in Alzheimer’s disease

**DOI:** 10.1002/alz.092439

**Published:** 2025-01-03

**Authors:** Ethan B Loew, Beth McCormick, John P Haran

**Affiliations:** ^1^ University of Massachusetts Chan Medical School, Worcester, MA USA

## Abstract

**Background:**

In Alzheimer’s disease (AD), changes in intestinal microbiota and systemic inflammation are concomitant with neuroinflammation and cognitive decline. This has led to the theory of microbial communities or infections as being causative in the development of neuroinflammation and immunosenescence seen in AD. Our research has demonstrated a decreased taxonomic diversity and an increased abundance of pathobionts in the gut of AD patients (Haran, *mBio* 2019), which is sufficient to promote amyloid and tau deposition in a mouse model (Chen, *Gut* 2023). It is unclear, however, if effects from the AD microbiome on the central nervous system can be mitigated. Here, we propose a protective mechanism where bacterial metabolites influence cognition *via* estrogen signaling.

**Method:**

We have performed gut microbiome profiling on a cohort of 178 nursing home residents and used *in vitro* models to quantify the effects of AD‐associated bacteria and bacterial products on inflammation.

**Result:**

We have identified a commensal bacterium, *Adlercreutzia equolifaciens*, to be depleted in our cohort of older adults with AD and further depleted in adults with progressive versus stable AD. *A. equolifaciens* metabolizes dietary isoflavones to produce (s)‐equol, a bacterial metabolite which binds to estrogen receptors. We treated cells expressing estrogen receptor beta, which is predominately expressed along the intestinal epithelium, with (s)‐equol and demonstrated (s)‐equol induces transcription of host genomic estrogen response genes (Vehicle normalized luciferase = 0.007, estradiol = 1.04, (s)‐equol = 1.23, p<0.0001). We have further demonstrated that treatment of intestinal epithelial cells with 17β‐estradiol induced the transcription of regulators of the innate immune response in an ERβ dependent manner (Vehicle RQ = 0.88, estradiol = 1.84, p<0.05). Finally, we have discovered that colonization with *A. equolifaciens* in the gut of older adults is associated with lower rates of colonization by the intestinal pathobiont *Escherichia* (Estimation of regression β‐coefficient = ‐1.422, p<0.05).

**Conclusion:**

*Adlercreutzia equolifaciens* and similar phytoestrogen‐metabolizing bacteria may protect the intestinal epithelium against bacterial invasion and subsequent systemic inflammation, thereby preventing cognitive decline. Our continuing work aims to further establish the connection between AD related neurocognitive decline, the microbiome, and immune system. *A. equolifaciens* is therefore a strong candidate as a microbiome‐based therapeutic acting along the gut‐brain axis in AD.

